# Greenhouse warming and anthropogenic aerosols synergistically reduce springtime rainfall in low-latitude East Asia

**DOI:** 10.1038/s41612-022-00295-x

**Published:** 2022-09-10

**Authors:** Young-Hee Ryu, Seung-Ki Min

**Affiliations:** 1 Division of Environmental Science and Engineering, Pohang University of Science and Technology (POSTECH), Pohang, South Korea; 2 Institute for Convergence Research and Education in Advanced Technology, Yonsei University, Incheon, Republic of Korea

**Keywords:** Climate and Earth system modelling, Climate-change impacts, Atmospheric dynamics

## Abstract

Low-latitude East Asia, particularly southern China, has experienced a markedly decreasing springtime rainfall in recent years whereas rainfall trends are weak in mid-latitude East Asia. Details of human influences on this contrasting feature remain uncertain. This study provides a quantification of the relative roles of greenhouse warming and aerosols in the observed spring rainfall trends over East Asia using a state-of-the-art numerical model. Greenhouse warming drives more rapid temperature increases over high-latitude East Asia potentially associated with reduced spring snow than the western North Pacific, which induces an anomalous anticyclone over the East China Sea. This circulation change results in a northwestward extension of the western North Pacific subtropical high, reducing rainfall at low latitudes while moderately increasing rainfall at mid-latitudes. In contrast, anthropogenic aerosols reduce rainfall in both low- and mid-latitude East Asia. Hence, the two anthropogenic factors synergistically reduce rainfall at low latitudes, with a stronger contribution of greenhouse warming (~34%) than aerosols (~17%). In mid-latitude East Asia, their contributions are offset, resulting in weak rainfall trends. Further, the anthropogenic influences are found to be relatively larger under drier conditions, suggesting that a more severe drought can occur in low-latitude East Asia under future drought-conducive conditions.

## Introduction

Spring rainfall plays a crucial role in agricultural production and food security and has critical socio-economic implications. In particular, spring rainfall in southern China has received considerable attention because of its unique characteristics. Spring rainfall in southern China is persistent from March to early May before the summer monsoon and accounts for ~35% of the annual rainfall^1^ with marked interannual variability. Several mechanisms have been proposed to explain its interannual variability including, the east-west thermal contrast between the Indochina Peninsula and the western North Pacific^2^, mechanical and thermal effects of the Tibetan Plateau^3^, anomalies of Eurasia snow^4,5^, and El Niño–Southern Oscillation variability^6–9^.

Compared to the interannual variability, long-term trends of spring rainfall have received less attention. A limited number of studies have reported a notable decreasing trend in spring rainfall in southern China^1,8,10,11^. The El Niño–Southern Oscillation is more likely to be responsible for the interannual variability of spring rainfall because of its periodic nature^7^. It has been demonstrated that intensified latent heating released by the convection over the South China Sea and Philippine Sea weakens the convergence of moisture flux and thus decreases rainfall in southern China^10^. However, the factors responsible for intensified deep convection in spring were not elucidated. Anthropogenic aerosols have been found to reduce April rainfall in southern China by ~14% through a comparison of numerical simulations under polluted and clean conditions using the Weather Research and Forecasting model with chemistry (WRF-Chem)^11^. However, aerosols in these simulations do not fully explain the 25%–33% reduction in observed rainfall over the past decades^8,11^. Zhang et al. examined the anthropogenic influences on the drought that occurred in southern China in May 2018 using large-ensemble climate model simulations^12^. They performed historical simulations with and without anthropogenic forcing and concluded that southern China’s 2018 late spring drought was primarily attributed to anthropogenic forcing. However, they did not identify the dominant anthropogenic factors; that is, greenhouse warming vs. aerosols. The previous studies^1,10,11^ that addressed decreasing trends in spring rainfall in southern China indicated that the decrease in spring rainfall is associated with a weakening of circulation accompanied by anomalous northeasterly flow in southern China, which reduces water vapor transport and rainfall. However, individual forcing contributions to long-term trends in weakening circulation and spring rainfall and the mechanisms driving the northeasterly anomalies remain unclear.

Therefore, the goals of this study are to: (1) assess trends of atmospheric circulation and rainfall in spring (March and April) over East Asia with a particular focus on southern China using the fifth-generation European centre for medium-range weather forecasts (ECMWF) reanalysis (ERA5) and observational rainfall (Global Precipitation Climatology Project, GPCP) data and (2) investigate whether the trends and their mechanisms are associated with human influences. Two factors are considered as anthropogenic forcings namely, temperature increase due to greenhouse gas increases (referred to as greenhouse warming) and anthropogenic aerosols. To quantify the individual contributions of these two factors, we employ the WRF-Chem model and carry out present-day simulations with high temperature (HIGH T) and current anthropogenic emissions (POLLUTED) and past-day simulations with low temperature (LOW T) and very low anthropogenic emissions (CLEAN) (see ‘Methods’). In addition to the WRF-Chem simulations, we utilize two Coupled Model Intercomparison Project Phase 6 (CMIP6) experiments^13^ that are forced by: (1) historical greenhouse gas changes only (hist-GHG) and (2) historical aerosol forcing changes only (hist-aer) for a robust understanding of the changes in atmospheric circulation and rainfall over East Asia.

## Results

### Observed changes in atmospheric circulation and rainfall

**Fig. 1:**
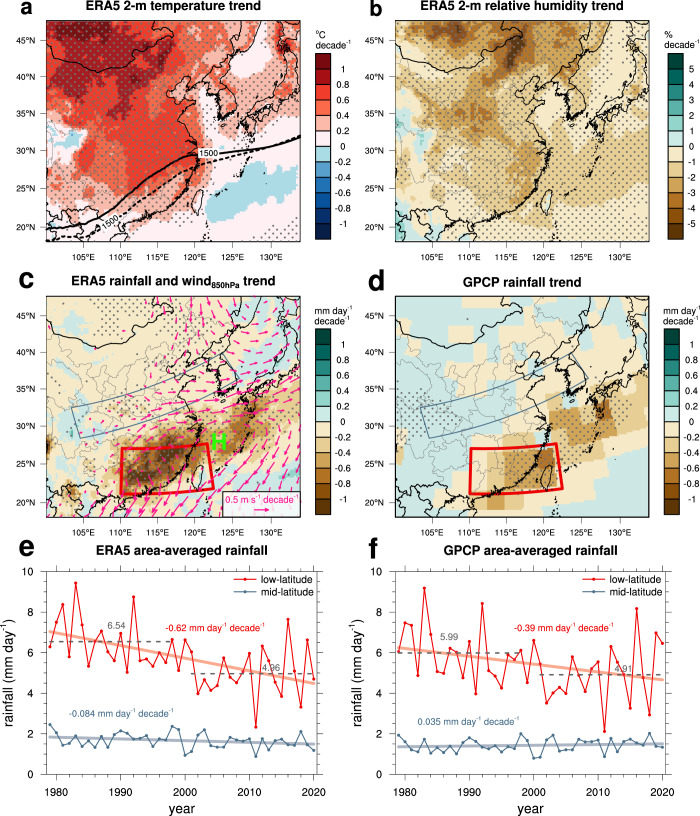
Observed trends in temperature, RH, rainfall and wind. ERA5 trends in March–April mean (**a**) temperature at 2 m and (**b**) relative humidity (RH) at 2 m during 1979–2020. In **a**, the thick-solid and thick-dashed lines indicate the March–April mean geopotential height of 1500 m at 850 hPa during the latter 20-year period (2001–2020) and the former 20-year period (1979–1998), respectively. **c** ERA5 trends in March–April mean daily rainfall (shading) and 925 hPa winds during 1979–2020. Regions where elevation is >800 m are masked out in the 925 hPa wind trends. The red rectangle indicates the low-latitudes (southern China) (22°–28°N, 110°–123°E), and the blue rectangle indicates the mid-latitudes. **d** GPCP rainfall trend during 1979–2020. The stipples in (**a**–**d**) indicate that the trends are significant at the 90% confidence level (*p*-value < 0.1). **e** Time series of March–April mean daily rainfall in the low-latitudes (red line with markers) and in the mid-latitudes (light blue with markers) and their linear trends (solid lines) from the ERA5 reanalysis data. **f** is the same as (**e**) but from the GPCP rainfall data. The numbers indicated above the gray-dashed lines in (**e**) and (**f**) are the 20-year mean daily rainfall for the 1979–1998 and 2001–2020 periods.

Spring temperature near the surface has increased during the last 42 years (1979–2020) over the majority of the East Asian continent (Fig. 1a), with an average rate of 0.40 °C decade^−1^ (average over the WRF-Chem domain). The increasing trend in temperature is most prominent in northern China and northwestern Mongolia (north of 45°N and west of 106°E) in spring, exhibiting an increasing trend of ~1 °C decade^−1^, which is comparable to the increasing temperature trend at high latitudes during 1982–2013^14^. The relative humidity (RH) exhibits significant decreasing trends in most regions with an average rate of −1.5% decade^−1^ (Fig. 1b), and the decreasing RH trends are at least partly associated with the increasing temperature trends. The decreasing RH trends thus can be partially responsible for the decreasing rainfall trends (Fig. 1c). However, the decreasing RH trends do not fully explain the decreasing rainfall trends in East Asia. For example, the RH trends in low latitudes are smaller than those in mid-latitudes but the decreasing rainfall trends are much larger in low latitudes than in mid-latitudes. For atmospheric circulation at 925 hPa, the wind trends clearly show northerly or northeasterly anomalies in southern China (Fig. 1c), as reported previously^1,10,12^. Since the southerly (southwesterly) wind prevails at 925 (850) hPa in southern China (Supplementary Fig. 1), the northerly or northeasterly anomalies indicate that the prevailing wind has weakened in recent decades, which appears to be associated with an extension of the western North Pacific subtropical high (bold contours in Fig. 1a). The boundary of the western North Pacific subtropical high extends northwestward in recent years (2001–2020) as compared to that in the past (1979–1998). Considerable decreasing rainfall trends are seen in low-latitude East Asia (Fig. 1c and d), for example, in southern China (22°–28°N, 110°–123°E) at an average rate of −0.62 mm day^−1^ decade^−1^ from the ERA5 (Fig. 1e) and −0.39 mm day^−1^ decade^−1^ from the GPCP data (Fig. 1f). The mean rainfall trend estimated from the ERA5 is consistent with those previously reported, e.g., −0.51 mm day^−1^ decade^−1^ obtained from five-reanalysis datasets^1^ and −0.56 mm day^−1^ decade^−1^ for April rainfall from the GPCP data^11^. The weaker trend from the GPCP data found in the present study than the ERA5 or from the previous study is possibly due to the higher rainfall from the ERA5 than the GPCP data (see climatology in Supplementary Fig. 2) or to the different periods/regions selected, respectively. Nonetheless, the overall spatial patterns of rainfall trends are similar between the two datasets. On the other hand, the rainfall trends in mid-latitudes are not as significant as those in low-latitudes in both datasets (the blue lines in Fig. 1e, f). The reasons for these weak trends are explored in a later section.

Together with the southwesterly-westerly trends at mid-latitudes (30°–35°N, 112°–122°E) (Fig. 1c), the low-level wind trends delineate an anomalous anticyclone centered approximately on the East China Sea (denoted by “H” in Fig. 1c). This anomalous anticyclone greatly influences water vapor transport in southern China because it counteracts the northeastward transport of water vapor (Supplementary Fig. 2 for the climatology of the vertically integrated water vapor flux). The prevailing southwesterly flow that brings water vapor northeastward from the South China Sea to southern China is one of the main channels supplying water vapor to this region^1,9^. Thus, the decrease in water vapor transport associated with the anomalous anticyclone is primarily responsible for the decreasing rainfall trend in southern China (Supplementary Fig. 2c). Similarly, the anomalous anticyclone explains the markedly decreasing rainfall trends over the East China Sea and southern Japan. Our result is consistent with that of a previous study^1^ which reported that the decreasing rainfall trend in southern China is primarily attributed to the weakening of dynamic components in the moisture budget, which means a weakening of the circulation.

### Effects of warming and aerosols simulated by WRF-Chem model

**Fig. 2:**
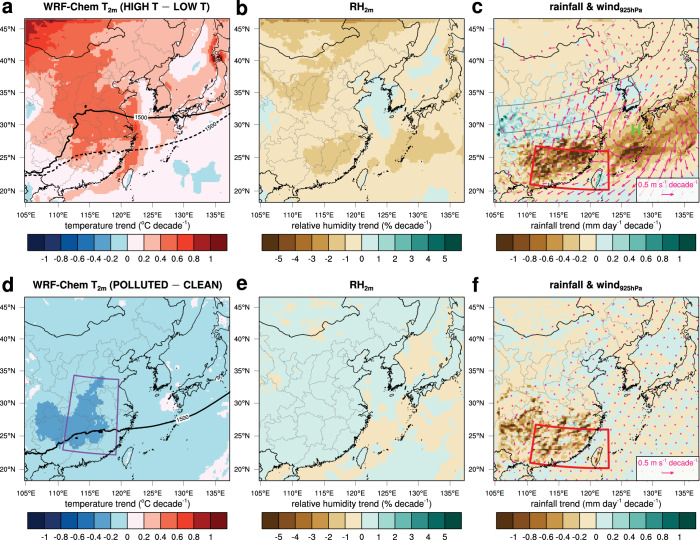
Effects of warming and aerosols from WRF-Chem simulations. Scaled trends in March–April mean (**a**) temperature at 2 m, (**b**) relative humidity at 2 m, **c** daily rainfall and 925 hPa winds. Note that the differences in the variables between the HIGH T_CLEAN and LOW T_CLEAN simulations in 2018 are divided by 50 years, and these values are referred to as the scaled trends. In **a**, the geopotential height of 1500 m at 850 hPa in the HIGH T_CLEAN (LOW T_CLEAN) simulation is highlighted by thick sold (dashed) line. **d**–**f** are the same as in (**a**–**c**) but compare HIGH T_POLLUTED and HIGH T_CLEAN simulations.

The effects of greenhouse warming are examined by comparing the HIGH T_CLEAN and LOW T_CLEAN simulations for March–April 2018 (Fig. 2a–c). The overall patterns of the temperature and RH trends between the two simulations are similar to those of the ERA5 temperature and RH trends, although the magnitudes are smaller in the WRF-Chem simulations than in the ERA5. Compared with the LOW T_CLEAN simulation, a northwestward extension of the western North Pacific subtropical high is reproduced in the HIGH T_CLEAN simulation (Fig. 2a). The low-level winds at 925 hPa also reveal northeasterly anomalies in low latitudes (Fig. 2c). In mid-latitudes (30°–35°N, 112°–122°E), southwesterly anomalies at 925 hPa are captured, and the anomalous flow is indicative of an anomalous anticyclonic circulation. Similarly, decreasing rainfall trends in southern China, the East China Sea, and southern Japan are found between the two simulations. For example, the difference in rainfall in southern China is 2.3 mm day^−1^ (−0.46 mm day^−1^ decade^−1^), which corresponds to a reduction of 40% (Table 1).

**Table 1:** March–April mean daily rainfall averaged over southern China in the WRF-Chem simulations for 2018 and 2019. The unit is mm day^−1^.

	LOW T (2018)	HIGH T (2018)	LOW T (2019)	HIGH T (2019)
CLEAN	5.84	3.53	10.7	7.57
POLLUTED	4.70	2.81	9.22	6.47

**Fig. 3:**
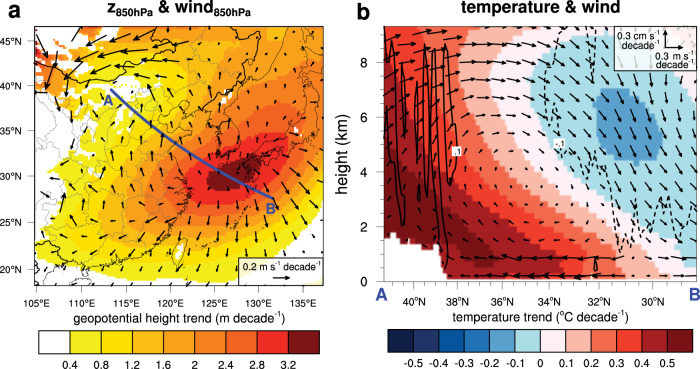
Thermal circulation induced by uneven temperature increases. **a** Scaled trends in March–April mean geopotential height and horizontal winds at 850 hPa computed with the HIGH T_CLEAN and LOW T_CLEAN simulations in 2018. **b** Vertical cross-sections of temperature (shading) and meridional-vertical winds (vectors) trends along line A–B shown in **a**. The solid (dashed) contours indicate the vertical wind trends of 0.1 (−0.1) cm s^−1^ decade^−1^. Note that the unit of vertical wind is cm s^−1^ decade^−1^ and that of meridional wind (positive northward) is m s^−1^ decade^−1^, and that the leftward is the northward in **b**.

The formation mechanism of the anomalous anticyclone is explored (Fig. 3). The geopotential height trend at 850 hPa between the HIGH T_CLEAN and LOW T_CLEAN simulations clearly shows an anomalous anticyclone centered over the East China Sea (31°N, 129°E), and the center is located slightly further northeast than the center observed in the ERA5. The center of the anticyclone is found in the downward branch of an anomalous thermal circulation induced by the temperature gradient in the northwest-southeast direction (line AB in Fig. 3a). As seen in the 2-m temperature trends in Fig. 2a, northwestern Mongolia exhibits the maximum rise in temperature. The vertical cross-section of the temperature trend (Fig. 3b) also shows larger warming at high latitudes (near point A). A higher temperature increase acts as an elevated heating source owing to the high elevation in the northwestern inland area. Therefore, anomalous upward flow is induced near point A, anomalous southerly flow in the lower atmosphere, returning northerly flow in the upper atmosphere, and downward flow over the East China Sea. Our results align with those of previous studies that examined the relationships between Eurasian snow and southern China rainfall^4,5^. A positive relationship between spring snow cover in western Siberia and spring rainfall in southern China has been found^4^. Zuo et al. showed that the decreasing snow water equivalent over high-latitude Eurasia since the late 1990 s corresponds to anomalous northeasterly flow and reduced rainfall in southeastern China^5^. The ERA5 snow depth trends also indicate large snow reductions in high-latitude Eurasia during 1979–2020 and shows anti-correlations with temperature (Supplementary Fig. 3). The Eurasian snow reduction in spring has been attributed to anthropogenic effects^15,16^ and greenhouse gas increases^17^.

To examine if the uneven temperature increases between high-latitudes and the western North Pacific are responsible for the changes in atmospheric circulations and rainfall in low latitudes, we additionally conduct a sensitivity simulation in which spatially uniform changes in temperature and RH are considered (CONST simulation, Supplementary Fig. 4; see ‘Methods’). It is found that rainfall especially in the low latitudes does not show significant decreasing trends and that circulation changes are not detectable (Supplementary Fig. 4e). Rather, slight decreases in rainfall are found in most regions in the HIGH T simulation, presumably due to the lower RH in the HIGH T simulation than in the CONST simulation. Therefore, our WRF-Chem simulations show that the rapid warming over high-latitude Eurasia associated with reduced snow owing to increasing greenhouse gases is highly likely responsible for the weakened atmospheric circulation and the rainfall reduction in low-latitude East Asia covering southern China, East China Sea, and southern Japan.

In contrast, the opposite result is found at mid-latitudes: the spring rainfall moderately increases due to the greenhouse warming, for example by 0.029 mm day^−1^ decade^−1^ in the region denoted by the blue rectangle (Fig. 2c). The GPCP data also show a weak increasing trend in the region at a rate of 0.035 mm day^−1^ decade^−1^ (the blue line in Fig. 1f). Several previous studies have also presented weak increasing trends of spring rainfall in central/central-western China from surface rain gauge observations^10,11^. Similarly, a future increase in spring rainfall in the mid-lower Yangtze River basin has been attributed to anthropogenic warming under the Representative Concentration Pathway (RCP) 8.5 scenario from Coupled Model Intercomparison Project Phase 5 (CMIP5) models^18^. The CMIP5 multi-model weighted mean precipitation in the previous study also exhibits decreased (increased) spring rainfall with anomalous northeasterly (southwesterly) flows in southeastern (central-eastern) China^18^. Such contrasting results between low- and mid-latitudes indicate an extension of the western North Pacific subtropical high, which pushes moisture transport northward or northwestward. A notable increasing trend in rainfall in northwest China during 1960–2010 has also been reported^19^, and the strengthening of the western North Pacific subtropical high has been suggested as one of the reasons for this trend.

The effects of aerosols on changes in circulation and rainfall are examined by comparing the HIGH T_POLLUTED and HIGH T_CLEAN simulations (Fig. 2d–f). It is found that higher aerosol loadings lead to lower temperatures in East Asia, as expected, and that the RH slightly increases over land due to the decreases in temperature. The cooling effects of aerosols have been commonly reported, which partly mask the human-induced warming^20^. Owing to aerosols, the decreasing temperature trend is largest in central China, with a trend as large as −0.31 °C decade^−1^ (Fig. 2d). However, minimal change in the atmospheric circulation is found (Fig. 2f). Hence, our WRF-Chem simulations suggest that the change in atmospheric circulation reported in the previous studies^1,10,11^ is primarily due to the greenhouse gas-induced warming rather than to aerosols.

**Fig. 4:**
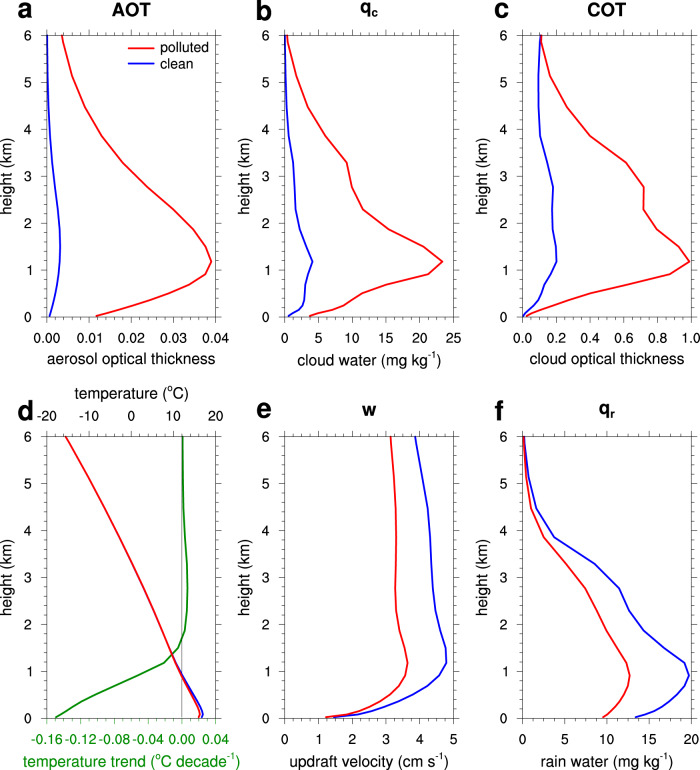
Impacts of aerosols in the vertical direction. March–April mean vertical profiles of (**a**) aerosol optical thickness (AOT) at 400 nm, (**b**) cloud water mixing ratio, (**c**) cloud optical thickness (COT), (**d**) air temperature (read the top axis) and scaled temperature trend (in green, read the bottom axis), (**e**) in-cloud updraft velocity, and (**f**) rain water mixing ratio in the HIGH T POLLUTED and HIGH T CLEAN simulations in 2018. All variables are averaged over central-east China (marked by the purple rectangle in Fig. 2d), and updraft velocity is averaged over cloudy grids at which the total-column cloud water mixing ratio is >10 mg kg^−1^. For AOT and COT, only daytime values are averaged.

Compared to clean conditions (HIGH T_CLEAN), a moderate decrease in rainfall is found in southern China with a trend of −0.144 mm day^−1^ decade^−1^ under polluted conditions (Fig. 2f and Table 1). Figure 4 shows the vertical profiles of area-averaged values and compares their changes due to aerosols over central-east China (24–36°N, 110–119°E, indicated in Fig. 2d). The massive anthropogenic pollutant emissions in the POLLUTED simulation cause large increases in aerosol optical thickness throughout the atmosphere (Fig. 4a). Because of the increases in aerosols that serve as cloud condensation nuclei, more cloud water droplets are formed, and so the cloud water mixing ratio largely increases by up to a factor 5 in the POLLUTED simulation (Fig. 4b). A large number of cloud water droplets results in increases in cloud optical thickness by a factor of 3 (Fig. 4c), and this considerably reduces solar radiation reaching the surface by 18% on average over central-east China. The reduced shortwave radiation in turn decreases the surface temperature and air temperature in the lower atmosphere (below ~1.7 km above ground level), contributing to increased atmospheric stability (Fig. 4d). The updraft velocity therefore is reduced, for example by 24% for the peak velocity (Fig. 4e), resulting in reductions in rainfall amounts (Fig. 4f). These are consistent with those previously found^11^, emphasizing the robustness of our findings.

It is noteworthy that greenhouse warming and anthropogenic aerosols both decrease spring rainfall in southern China and that the effect of greenhouse warming is approximately twice (−40% in spring 2018) larger than that of aerosols (−20% in spring 2018) (Table 1). The relative decrease in rainfall due to aerosols in our simulations is comparable to that obtained from the previous WRF-Chem simulations (14% for April 2009)^11^. These results further highlight that the effects of greenhouse warming should be considered when explaining the notable reductions observed in spring rainfall.

Unlike the temperature increase, high aerosol loadings reduce rainfall in mid-latitudes (−0.040 mm day^−1^ decade^−1^, Fig. 2f). Thus, these results imply that the weak rainfall trends found in the observation and reanalysis at mid-latitudes result from the offsetting effects of greenhouse warming and aerosols. The weak decreasing rainfall trends observed in some regions at mid-latitudes can be interpreted as the larger contribution of aerosols than that of greenhouse warming.

Another important finding is that the two factors exert relatively larger influences when the atmospheric conditions are drier (2018) than wetter (2019) (Table 1), although the mechanism and results are generally similar between 2018 and 2019 (cf. Fig. 2 and Supplementary Fig. 5). The relative decreases in rainfall in southern China due to greenhouse warming are −40% for spring 2018 and −29% for spring 2019. Moreover, those due to aerosols are −20% for spring 2018 and −15% for spring 2019. This finding is also supported by the further northwestward extension of the western North Pacific subtropical high in 2018 compared to that in 2019 (cf. Fig. 2a and Supplementary Fig. 5a). It should be noted that the absolute differences between the simulations are slightly larger in 2019 than in 2018, so the larger influences of two anthropogenic factors should be interpreted in a relative sense. Nonetheless, our results imply that greenhouse warming and high anthropogenic aerosol loadings pose a higher risk of drought in low-latitude East Asia under drier atmospheric conditions.

### Comparison of WRF-Chem simulations with CMIP6 experiments

**Fig. 5:**
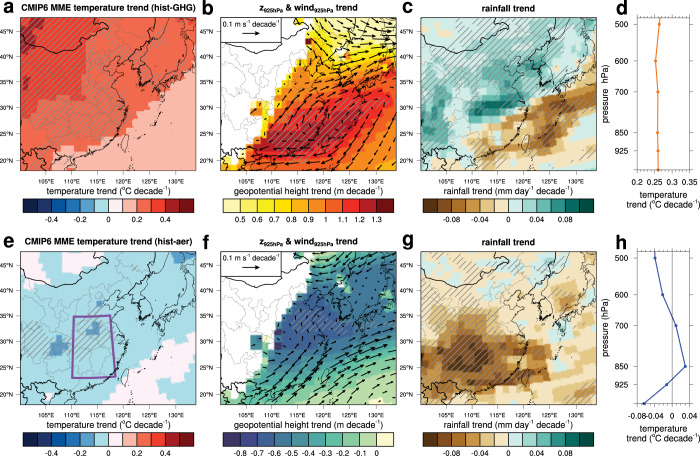
CMIP6 hist-GHG and hist-aer experiment results. Multi-model ensemble (MME) March–April mean (**a**) temperature trend, (**b**) geopotential height and winds at 925 hPa trends, (**c**) daily rainfall trend during 1979–2020, and (**d**) vertical profile of temperature trend averaged over central-east China (marked by the purple rectangle in **e**) in the CMIP6 hist-GHG experiment. Note that the temperature trend at the lowest *y*-axis corresponds to that at 2 m. **e**–**h** are the same as in **a**–**d** but in the CMIP6 hist-aer experiment. The slanted lines in **a**/**e** indicate the regions where the increasing/decreasing trends of near-surface temperature are larger/smaller than 0.2/−0.05 °C decade^−1^ are found in at least five out of seven models. The slanted lines in **b**/**f** indicate the regions where the increasing/decreasing trends of geopotential height at 925 hPa are larger/smaller than 1.1/−0.4 m decade^−1^ are found in at least five out of seven models. The regions showing the same sign of increasing or decreasing trends in rainfall in at least five out of seven models are marked by the slanted lines in **c** and **g**.

The multi-model ensemble (MME) means in the hist-GHG experiments generally show consistent results with those observed in the ERA5 and WRF-Chem simulations (Fig. 5a–d): a notable increasing trend of temperature in northwest Mongolia relative to the western North Pacific, anomalous anticyclone and northeasterly/easterly anomalies over the western North Pacific, and decreasing rainfall trends in low-latitude East Asia. The magnitudes of the MME mean trends are much smaller than those found in the reanalysis, presumably because of the weaker increasing trends in temperature and canceling-out effects among the individual models. For example, the centers of anomalous anticyclones greatly vary among the models (highlighted by “H” in Supplementary Fig. 6). It should be noted that the seven models that capture the notable increasing trend of temperature in northwest Mongolia relative to that in the western North Pacific (see’Methods’) commonly show anomalous anticyclonic circulations that are centered on either inland in southern China or the western North Pacific. In these models, rainfall shows decreasing trends in the regions near or influenced by the anticyclonic circulations. On the other hand, the remaining three models do not explicitly show such anticyclonic circulations within the study domain (to the south of ~32°N and to the west of ~128°E).

Moreover, the results from the his-aer experiments (Fig. 5e–f) are generally consistent with those found in the WRF-Chem simulations (POLLUTED minus CLEAN simulations, Figs. 2d and f, 4). The high aerosol loadings cool the surface (Fig. 5e); the degree of cooling is largest near the surface and decreases with height; and then aerosols act to slightly warm the atmosphere near 850 hPa level (Fig. 5h). The warming above the boundary layer can be attributed to the warming by black carbon aerosols^21,22^. The increased atmospheric stability due to aerosols (Fig. 5h) therefore depresses rainfall over most of East Asia (Fig. 5g). The wind trends however do not show a direct link to the circulation changes that are observed in the reanalysis data. The southwesterly trends over southern China are likely because the results of some models that show anomalous cyclones due to cooling by aerosols (highlighted by “L” in Supplementary Fig. 7 for five out of ten models) are reflected in the MME mean. When all the ten models are taken into account (Supplementary Fig. 8), the decreasing temperature trends over central-east China become stronger, the southwesterly trends become weaker and the vertical profile of temperature trend becomes similar to that is found in the WRF-Chem simulations (Fig. 4d). These results imply that models’ responses to aerosols are complex and have a very broad spectrum^20^.

Additionally, much more significant increasing trends in spring rainfall at mid-to-high latitudes are found in the hist-GHG experiment (Fig. 5c) as compared to the WRF-Chem simulations, which has been also reported in the CMIP5 RCP8.5 scenario^18^. This can be interpreted as an extension of the western North Pacific subtropical high inferred from the anomalous anticyclonic circulation (Fig. 5b). Given that high aerosol loadings reduce rainfall over the majority of the domain in the hist-aer experiment, consistent results between the WRF-Chem simulations and CMIP6 experiments are obtained. This CMIP6-based assessment largely supports that the greenhouse warming and aerosols both contribute to decreasing spring rainfall at low latitudes, and the contribution of aerosols that decreases rainfall offsets the influence of greenhouse warming that increases rainfall at mid-to-high latitudes.

## Discussion

We explored the individual effects of greenhouse warming and aerosols on changes in atmospheric circulation and rainfall in spring over East Asia through WRF-Chem simulations. The reanalysis data indicate increasing trends in temperature over East Asia, with a particularly rapid increasing trend over northwestern Mongolia. The northeasterly anomalies in southern China counteract the northeastward water vapor transport and thus decrease rainfall in spring. The WRF-Chem simulation results are summarized as follows:

1. Through a comparison of the control simulation and sensitivity simulation in which increasing temperature trends are removed, an anomalous anticyclonic circulation over the East China Sea and a northwestward extension of the western North Pacific subtropical high are found as a result of a thermal circulation that is induced by the higher temperature increase in northwestern Mongolia relative to that over the western North Pacific.
2. The northeasterly anomalous flow associated with the anomalous anticyclone counteracts the water vapor transport from the South China Sea and hence decreases spring rainfall at low latitudes (southern China, the East China Sea, and southern Japan).
3. Simultaneously, the anomalous southwesterly flow at mid-latitudes due to the anomalous anticyclone moderately increases spring rainfall at mid-latitudes.
4. Aerosols have minimal impact on the change in atmospheric circulation but moderately decrease rainfall in both low- and mid-latitude continents by increasing atmospheric stability.
5. Thus, the effects of greenhouse warming and aerosols on rainfall act in the same direction at low latitudes, which significantly decrease rainfall in southern China by 29–40% and 15–20% for the specific years simulated, respectively; however, their effects are exerted in the opposite direction at mid-latitudes. In southern China, the contribution of greenhouse warming to decreasing rainfall is twice than that of aerosols.
6. The anthropogenic influences on spring rainfall in southern China are larger when atmospheric conditions are drier in a relative sense.

The results of CMIP6 hist-GHG and hist-aer experiments are consistent in general with those of the WRF-Chem simulations. In the hist-GHG experiments, spring rainfall decreases at low latitudes but increases at mid-high latitudes. The CMIP6 models that capture the strongly increasing temperature trends in northwestern Mongolia produce anomalous anticyclonic circulations over southern China or the western North Pacific. This confirms that the rapid temperature increase over Eurasia in spring is likely responsible for the formation of the anomalous anticyclone. The decrease in rainfall due to aerosols is noted over most East Asia in the hist-aer experiment, supporting that the combined effects of greenhouse warming and aerosols significantly decrease spring rainfall at low latitudes (e.g., southern China). On the other hand, the offsetting effects of greenhouse warming and aerosols at mid-latitudes lead to weak trends in spring rainfall. It is generally agreed that the regional effects of aerosols on temperature, circulation and precipitation are sensitive to a number of model uncertainties, inducing large inter-model differences^20^; hence, multi-model approaches would be required for a robust understanding of the climate responses to regional aerosol forcings^23^. Nevertheless, our results based on WRF-Chem and CMIP6 multi-model simulations support the important role of anthropogenic aerosols in shaping spring drying over low-latitude East Asia.

It is noteworthy that although the ERA5 winds at 850 hPa show the northeasterly trends over southern China and the East China Sea, the vertical winds at 600 hPa do not clearly exhibit upward/downward trends associated with an anomalous anticyclonic circulation encompassing Mongolia and the East China Sea (Supplementary Fig. 9). We speculate that there could be other mechanisms that can interplay or interfere with the mechanism we suggested. One example is the heat-induced anomalous overturning circulation reinforced by tropical convection over the Philippine Sea^10^. Therefore, further comprehensive studies are warranted.

East Asian temperatures are projected to rise continuously in the near future in all emission scenarios with larger warming magnitudes in its northern part^24^, which would strengthen the southern drying-northern wetting trends in spring. However, anthropogenic pollutant emissions are projected to decrease over time^25^, which can alleviate the drying trend at low-latitudes but intensify the wetting trend at mid-latitudes to some extent. The latter is in line with a recent study that showed a considerable increase in June-July rainfall in eastern China due to abruptly reduced aerosols during the COVID-19 pandemic^26^, indicating more chances of floods at mid-latitudes. In this respect, further studies are needed to better predict the combined influences of greenhouse warming and aerosol reduction on the near future spring hydrology in East Asia. Changing spring rainfall in warmer climates imply that more attention should be paid to increasing risk of hydro-meteorological extremes and sustainable water management over East Asia.

## Methods

### Reanalysis and observation data

The ERA5 data at 0.25° × 0.25° resolution for March and April 1979–2020 are used^27^. The original ERA5 data are available at hourly intervals, but we utilize the dataset at 3-h intervals and compute March–April mean values of near-surface temperature, relative humidity, daily rainfall, geopotential height, and horizontal winds for each year during 1979–2020. Linear regression analyses are performed for all grids within the study domain (Fig. 1a–c). The 20-year mean values of 2-m temperature, rainfall, 925 hPa winds, 850 hPa geopotential height and winds during the 1979–1998 and 2001–2020 are compared (Supplementary Fig. 1). The former and latter 20-year periods are selected because the 20-year mean rainfall values over southern China are significantly different between the two periods (Fig. 1e, f). For ERA5 rainfall, it is 4.96 mm day^−1^ during 2001–2020 and 6.54 mm day^−1^ during 1979–1998 with a *p*-value of 0.0025 (calculated with a two-sided Wilcoxon signed-rank test). The GPCP version 2.3 monthly precipitation data at 2.5° × 2.5° resolutions^28^ are used to compute spring rainfall trends during the same period used in the ERA5 trend computation (Fig. 1d and f). Similar results are found for the GPCP rainfall for the two periods with a *p*-value of 0.03. In addition to the trends shown in Fig. 1, the trend of sea surface temperature (SST) are computed, and these trends are used in the WRF-Chem sensitivity simulations.

We use the HadCRUT5 gridded data of global historical surface temperature anomalies^29^, version 5.0.1.0 to assess the long-term temperature trend over East Asia. For gridded daily temperature, the NOAA/NCEP Climate Prediction Center (CPC) global temperature data at 0.5° × 0.5° resolutions are utilized to evaluate the WRF-Chem performance in reproducing near-surface temperature for the 2018 and 2019 control simulations. The daily CPC minimum and maximum temperatures are averaged to represent daily mean temperature, and the same computation is applied to the simulated temperatures. For rainfall validation, the global historical climatology network (GHCN) daily precipitation data are used^30^. The stations located within the study domain (18–48°N, 105–142°E) are selected, and the number of stations used in the validation is 275.

### CMIP6

Two CMIP6 experiments are employed in this study: one forced by historical greenhouse gas concentrations only (hist-GHG) and the other forced by historical changes in anthropogenic aerosol forcing (hist-aer)^13^. Initially, we considered ten models that fulfilled hist-GHG and hist-aer experiments and had data available during 1979–2020 with at least three ensemble members. However, three out of ten models are excluded from the analysis (Supplementary Table 1) because the models do not reproduce or underrepresent the spatial patterns of increasing temperature trends found in the ERA5 (Supplementary Fig. 10 for the ERA5 temperature trends, and Supplementary Fig. 6 for the CMIP6 models’ trends). We select the seven CMIP6 models that reproduce the northwest-southeast gradient of increasing temperature trend with a slope >10% of the ERA5. Hence, a total ensemble with 43 members are used in our analysis. It is worth noting that our selection of models based on their skill in simulating the past climate might lead to the omission of the possible pathways that could have occurred in the real world, but the results using the models with sufficient skill would be more representative of real-world responses to greenhouse warming. The variables considered are the monthly near-surface temperature, rainfall, geopotential height, and horizontal winds. Analogous to the ERA5, March–April mean values and trends during 1979–2020 are computed and analyzed.

### WRF-Chem modeling

The WRF-Chem model version 4.1.2, in which wet and dry deposition of aerosols are recently updated by Ryu and Min^31^, is used in this study. The updated WRF-Chem model shows satisfactory performance in reproducing surface PM_2.5_ and PM_10_ concentrations as well as wet deposition fluxes of soluble ions over East Asia^31^. The horizontal grid spacing is 20 km, and the model domain is shown in Fig. 2. Note that the WRF-Chem domain is set to be slightly smaller than the domain used for ERA5 and CMIP6 analyses due to computational constraints. We consider two cases for spring 2018 and 2019, regarded as a dry and a wet year, respectively, based on the ERA5 rainfall reanalysis. These two cases are selected to examine if the two anthropogenic factors exert consistent influences on spring rainfall for a dry and a wet year. The March–April mean daily rainfall averaged over southern China (22°–28°N, 110°–123°E, depicted by the red rectangle in Fig. 1c) is 3.32 mm day^−1^ in 2018 and 6.63 mm day^−1^ in 2019, and its 20-year mean (±standard deviation) during 2001–2020 is 4.96 ± 1.22 mm day^−1^. All WRF-Chem simulations are initialized at 15 UTC on February 24 with a 4-day spin-up. The ERA5 data are used as the initial and boundary conditions at 3-h intervals for meteorology. The WRF-Chem simulations are re-initialized every four days. We use Morrison two-moment bulk scheme^32^ as a microphysics scheme with prognostic cloud condensation nuclei-activation. The activation of aerosols is parameterized following Abdul-Razzak and Ghan^33^. The complete physical and chemical options/parameterizations are listed in Supplementary Table 2, and the detailed WRF-Chem configurations are described in Ryu and Min^31^.

To examine the effects of greenhouse warming and anthropogenic aerosols separately, we designed one control and three sensitivity WRF-Chem simulations. Four simulations are required to examine the individual contributions of two factors^34^ separately. The control simulation represents the current meteorology/climate and pollutant emissions; therefore, no modifications are made (referred to as the HIGH T_POLLUTED simulation). We regard the 50-year ago meteorology as past meteorology, and so the ERA5 trends of temperature, SST, and RH are extrapolated, which are then subtracted from the current temperature, SST, and RH, respectively, at all the grids (only surface grids for SST). We adopt the detrending approach to examine the effects of greenhouse warming. The 50-year period is chosen in this study because the temperature increase has accelerated since ~1970 s^35^ (also see Supplementary Fig. 11). This recent rapid warming has been robustly attributed to the anthropogenic influences^36^. Analysis nudging is applied above the boundary layer in all simulations so that the temperature decrements can be maintained throughout the sensitivity simulations. The sensitivity simulations in which the air temperature and SST are lower than the current values are called LOW T simulations. The other meteorological variables (e.g., wind and geopotential height) are not altered in all the simulations. All anthropogenic pollutant emissions are reduced 50 times, and the initial and boundary conditions for all pollutants except ozone are accordingly reduced 50 times to consider the low concentrations of aerosols and precursor gases in the past. The 50 times reductions in nitrogen oxides and sulfur dioxide emissions, for example, in China roughly correspond to the emissions in 1951 and 1950, respectively, based on the historical anthropogenic emission estimations^37^. The natural emissions in the sensitivity simulations do not change. Simulations with reduced anthropogenic pollutant emissions are called CLEAN simulations. Therefore, the conducted WRF-Chem simulations are the HIGH T_POLLUTED, LOW T_POLLUTED, HIGH T_CLEAN, and LOW T_CLEAN simulations (Table 1).

The simulated near-surface temperature and rainfall in the control simulation (HIGH T_POLLUTED simulation) are evaluated against the CPC global temperature data and the GHCN daily precipitation data, respectively (Supplementary Figs. 12 and 13). Note that the simulated temperature at 20 km × 20 km resolutions is regridded onto the CPC grids (0.5° × 0.5°). The WRF-Chem model shows reasonable performance in reproducing temperature (e.g., for 2018 a mean bias of 0.07 °C, an RMSE of 1.62 °C, and a spatial correlation of 0.99 over land grids) and precipitation (a mean bias of −0.45 mm day^−1^, an RMSE of 2.06 mm day^−1^ and a correlation coefficient of 0.61 averaged over the observation stations).

We conduct an additional simulation to examine if the larger degree of temperature increases due to (potentially) larger snow melting at high latitudes is responsible for the decreases in spring rainfall at low latitudes. Because it is hard to directly control the amount of snow and the degree of snow melting within the WRF-Chem model, we consider spatially uniform changes in temperature and RH in the additional sensitivity simulation (hereafter, CONST simulation). So, the domain-averaged warming and drying trends obtained from the ERA5 reanalysis data are subtracted from the current meteorological fields in all the vertical layers in the CONST simulation (i.e., their spatial distributions are eliminated) (Supplementary Fig. 4). In other words, the CONST simulation also assumes lower temperature and higher RH relative to the present ones, so that the vertical profiles of domain-averaged temperature and RH in the CONST simulation are similar to those in the LOW T simulation (Supplementary Fig. 4a and b).

## Supplementary information

Supplementary Information

## Data Availability

The pressure-level ERA5 data are freely available from https://cds.climate.copernicus.eu/. The GPCP version 2.3 rainfall data can be downloaded from https://psl.noaa.gov/data/gridded/data.gpcp.html. The HadCRUT5 data were downloaded at https://crudata.uea.ac.uk/cru/data/temperature/#datdow. The CPC Global Temperature data provided by the NOAA/OAR/ESRL PSD, Boulder, Colorado, USA, were obtained from the website at https://psl.noaa.gov/data/gridded/data.cpc.globaltemp.html. The GHCN precipitation data are available at https://www.ncei.noaa.gov/products/land-based-station/global-historical-climatology-network-daily. We obtained the CMIP6 data from https://esgf-node.llnl.gov/search/cmip6/. The public version of WRF-Chem source code is available from https://github.com/wrf-model/WRF/releases/tag/v4.1.2. The updated wet and dry deposition schemes for aerosols can be found in 10.5281/zenodo.5895233. The WRF-Chem outputs are available upon request from Y.-H.R.

## Code availability

The NCAR Command Language (NCL) scripts that are used for analyses and plotting the figures can be available upon request from Y.-H.R.
