# Supplementary material for: Greenhouse warming and anthropogenic aerosols synergistically reduce springtime rainfall in low-latitude East Asia
Source: NPJ Clim Atmos Sci. 2022 Sep 10;5(1):69. doi: 10.1038/s41612-022-00295-x (PMC9463665; doi:10.1038/s41612-022-00295-x)
Supplement: Supplementary file 1 — Supplementary Information [file 41612_2022_295_MOESM1_ESM.pdf]

Supplementary Information

Greenhouse warming and anthropogenic aerosols synergistically reduce springtime rainfall in low-latitude East Asia

Young-Hee Ryu[1], Seung-Ki Min[1,2]

[1]Division of Environmental Science and Engineering, Pohang University of Science and Technology (POSTECH), Pohang, South Korea

[2] Institute for Convergence Research and Education in Advanced Technology, Yonsei University, Incheon, Republic of Korea

Corresponding author: Seung-Ki Min (skmin@postech.ac.kr), 77 Cheongam-ro, Pohang 37673, South Korea.

Phone: +82-(0)54-279-2286

Fax: +82-(0)54-279-8299

Contents of this file

Supplementary Tables 1 to 2

Supplementary Figures 1 to 13

Supplementary References

Supplementary Table 1. CMIP6 models considered in the present study, and the seven models used in the analysis are highlighted by bold.

Model name	Modeling center	Ensemble members
**ACCESS-ESM1-5**	Commonwealth Scientific and Industrial Research Organisation (CSIRO), Australia	r[1–3]i1p1f1
**BCC-CSM2-MR**	Beijing Climate Center, China	r[1–3]i1p1f1
**CanESM5**	Canadian Centre for Climate Modelling and Analysis, Canada	r[1–15]i1p1f1
**CNRM-CM6-1**	Centre National de Recherches Meteorologiques (CNRM), France	r[1–10]i1p1f2
FGOALS-g3	Chinese Academy of Science (CAS), China	r[1–3]i1p1f1
**HadGEM3-GC31-LL**	Met Office Hadley Centre, United Kingdom	r[1–4]i1p1f3
IPSL-CM6A-LR	Institut Pierre Simon Laplace, France	r[1–10]i1p1f1
**MIROC6**	Japan Agency for Marine-Earth Science and Technology, Japan	r[1–3]i1p1f1
**MRI-ESM2-0**	Meteorological Research Institute, MRI	r[1–5]i1p1f1
NorESM2-LM	Norwegian Meteorological Institute, Norway	r[1–3]i1p1f1

Supplementary Table 2. WRF-Chem modeling configurations

Description/Scheme	Selection
Horizontal grid size (number of grids, nx×ny)	20 km (185×170)
Number of vertical layers (top pressure)	40 (40 hPa)
Time step	60 s
Initial/boundary conditions (updated intervals)	ERA-5 (3-hourly)
Coefficients used in Four-Dimensional Data Assimilation (FDDA)	3×10^{-4} s^{-1} for wind and temperature<br>5×10^{-5} s^{-1} for moisture
FDDA calculation frequency	60 s
Shortwave and Longwave radiation	RRTMG[1]
Boundary layer	MYNN[2]
Land surface	Noah[3]
Microphysics	Morrison two moment[4]
Cumulus	Grell-Freitas[4]
Gas-aerosol chemistry	MOZART-MOSAIC[5,6]
Aerosol activation	Abdul-Razzak and Ghan[7]
Aqueous chemistry for resolved clouds	Fahey and Pandis[8]
Aqueous chemistry for subgrid clouds	Walcek and Taylor[9]
Photolysis	Updated TUV[10]
Dry deposition for gas	Wesely[11]
Dry deposition for aerosols	Binkowski and Shankar[12]
Wet deposition for resolved clouds/rain	Ryu and Min[13]
Wet deposition for subgrid clouds/rain	Grell and Devenyi[14]
Initial/boundary conditions for atmospheric composition (updated intervals)	CAMS[15] (3-hourly)
Anthropogenic emissions	REASv3.2[16]
Biogenic emissions	MEGANv2.04[17]

Biomass burning emissions	GFASv1.2[18,19]
Dust/sea salt emissions	GOCART[20–22]

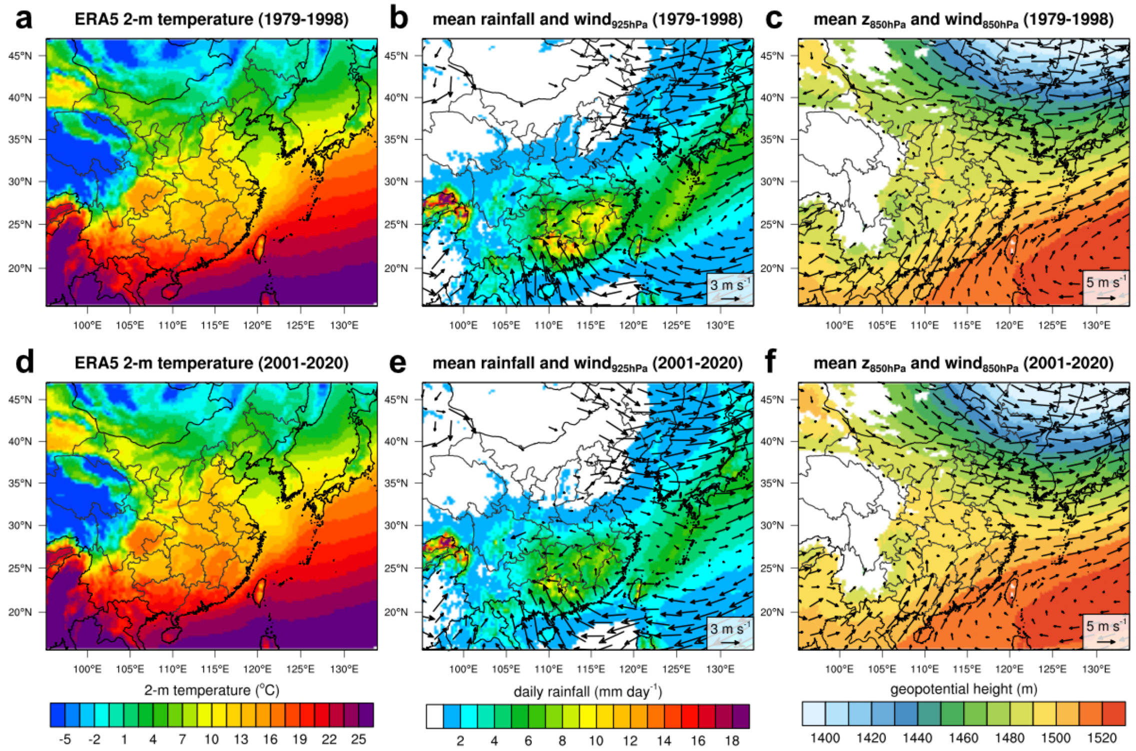

Supplementary Figure 1. 20-year mean temperature, rainfall, wind, and geopotential height. ERA-5 reanalysis (a) March-April mean 2-m temperature, (b) mean daily rainfall and winds at 925 hPa, and (c) mean geopotential height and winds at 850 hPa averaged for 1979–1998. (d–f) are the same as in (a–c) but for 2001–2020.

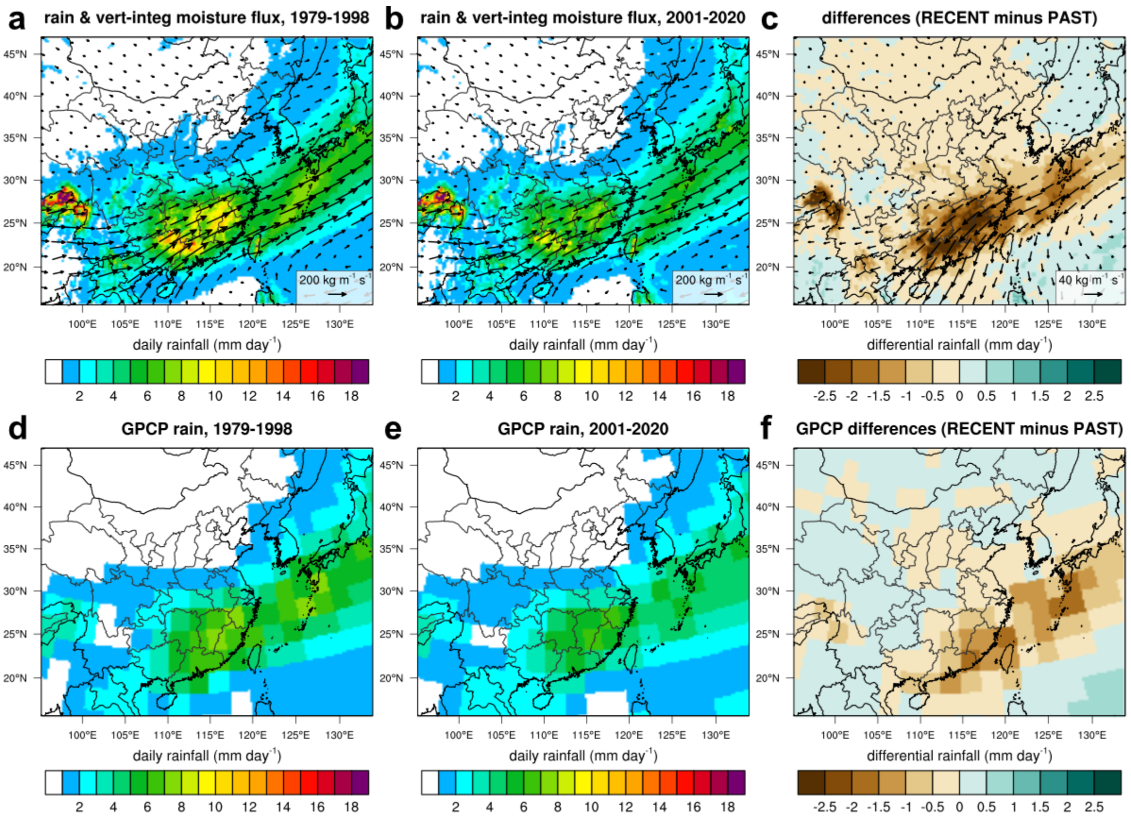

Supplementary Figure 2. ERA5 and GPCP 20-year mean rainfall. ERA5 March-April mean daily rainfall (shading) and vertically-integrated moisture flux (vectors) during (a) the past 20 years (1979–1998) and (b) the recent 20 years (2001–2020). (c) Differences in daily rainfall and vertically-integrated moisture flux between the recent and past periods. GPCP March-April mean daily rainfall during (d) the past 20 years (1979–1998) and (e) the recent 20 years (2001–2020). (f) Difference in daily rainfall, recent 20-year mean minus past 20-year mean.

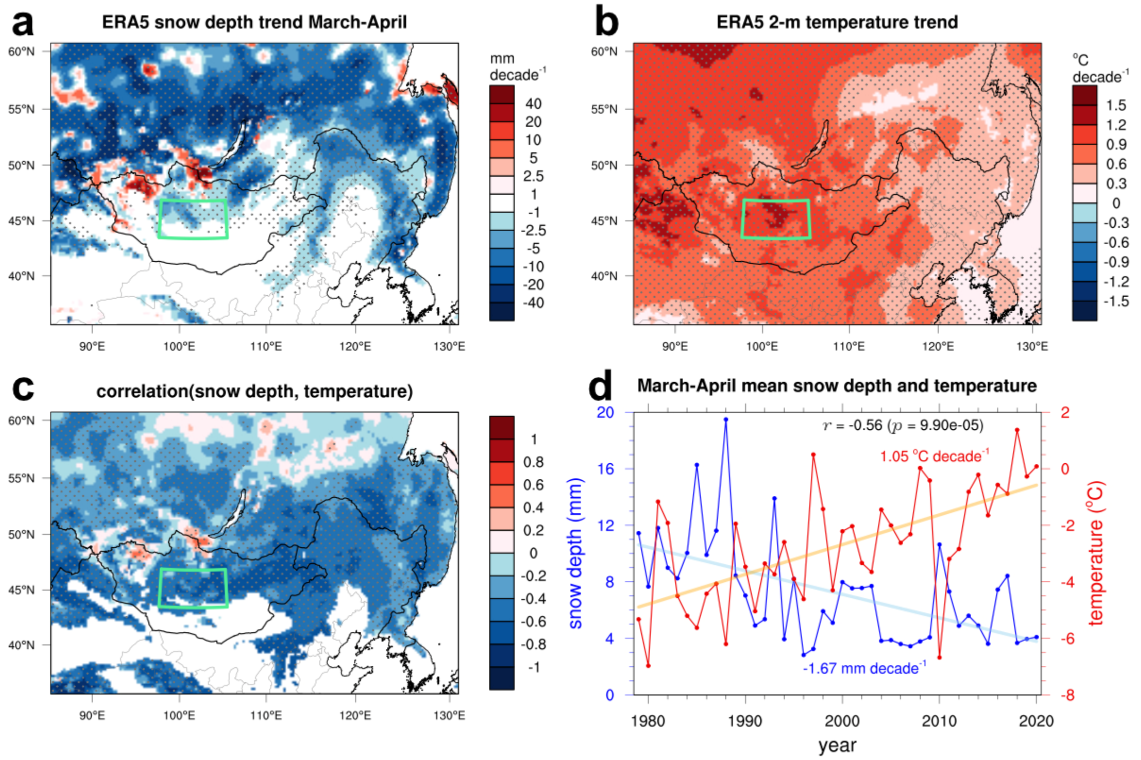

Supplementary Figure 3. Trends in snow depth and temperature. ERA5 trends of March-April mean (a) snow depth (mm decade^{-1}) and (b) temperature at 2 m (°C decade^{-1}) computed by a linear regression method during 1979–2020. (c) Linear correlation coefficients between snow depth and 2-m temperature. In (a and c), only grids in which the 42-year mean snow depth is greater than 1 mm are shown, and the stipples indicate that the trends or correlations are significant at the 90% confidence level (p-value < 0.1). (d) Time series of snow depth (blue) and 2-m temperature (red) averaged over central Mongolia (45°–48.5°N, 97°–°106E, denoted by the green box in (a–c)).

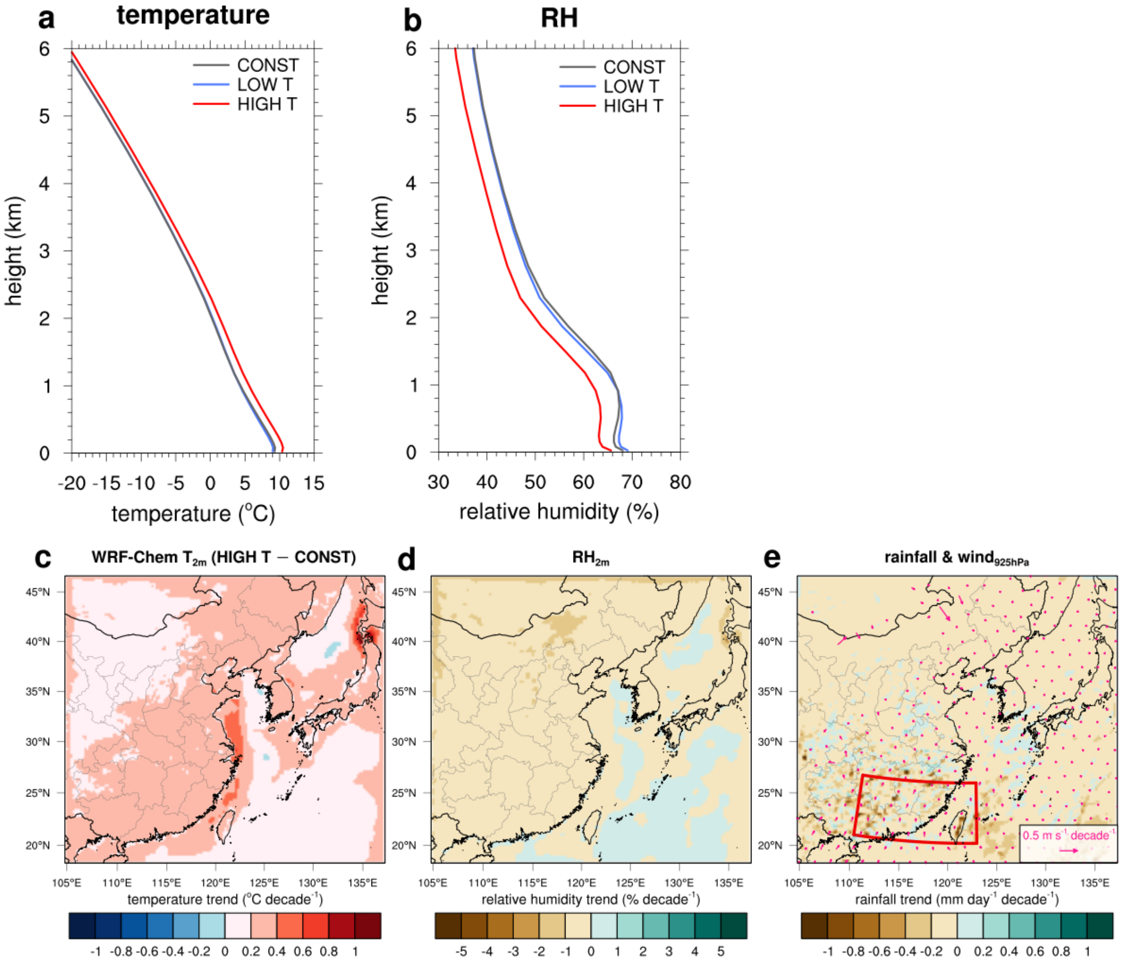

Supplementary Figure 4. WRF-Chem sensitivity simulation with constant temperature and RH trends. (a and b) Vertical profiles of domain-averaged temperature and relative humidity (RH), respectively, in the HIGH T, LOW T, and CONST simulations. Scaled trends in March-April mean (c) temperature at 2 m, (d) RH at 2 m, and (e) rainfall and horizontal winds at 925 hPa between the HIGH T and CONST simulations.

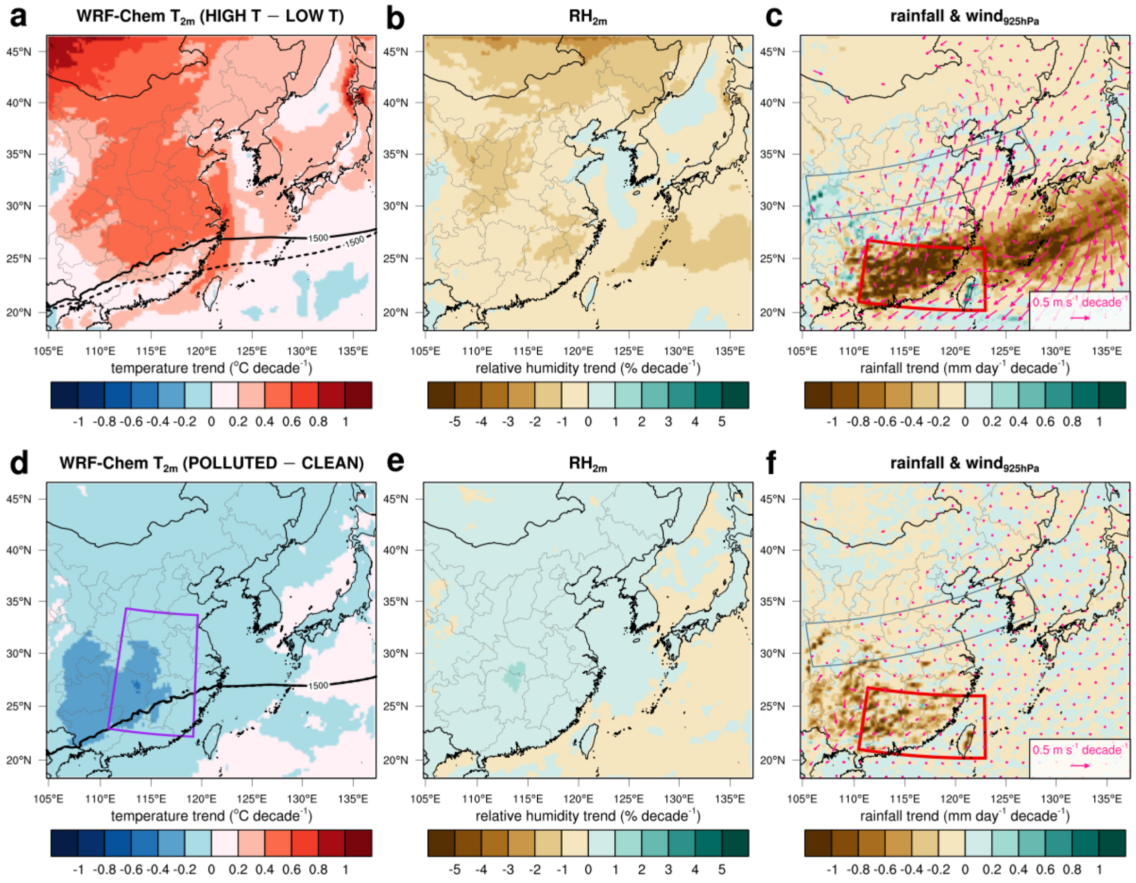

Supplementary Figure 5. Effects of warming and aerosols from 2019 WRF-Chem simulations. Scaled trends in March-April mean (a) temperature at 2 m, (b) relative humidity at 2 m, (c) daily rainfall and 925 hPa winds. Note that the differences in the variables between the HIGH T_CLEAN and LOW T_CLEAN simulations in 2019 are divided by 50 years, and these values are referred to as the scaled trends. In (a), the geopotential height of 1,500 m at 850 hPa in the HIGH T_CLEAN (LOW T_CLEAN) simulation is highlighted by thick sold (dashed) line. (d–f) are the same as in (a–c) but compare HIGH T_POLLUTED and HIGH T_CLEAN simulations.

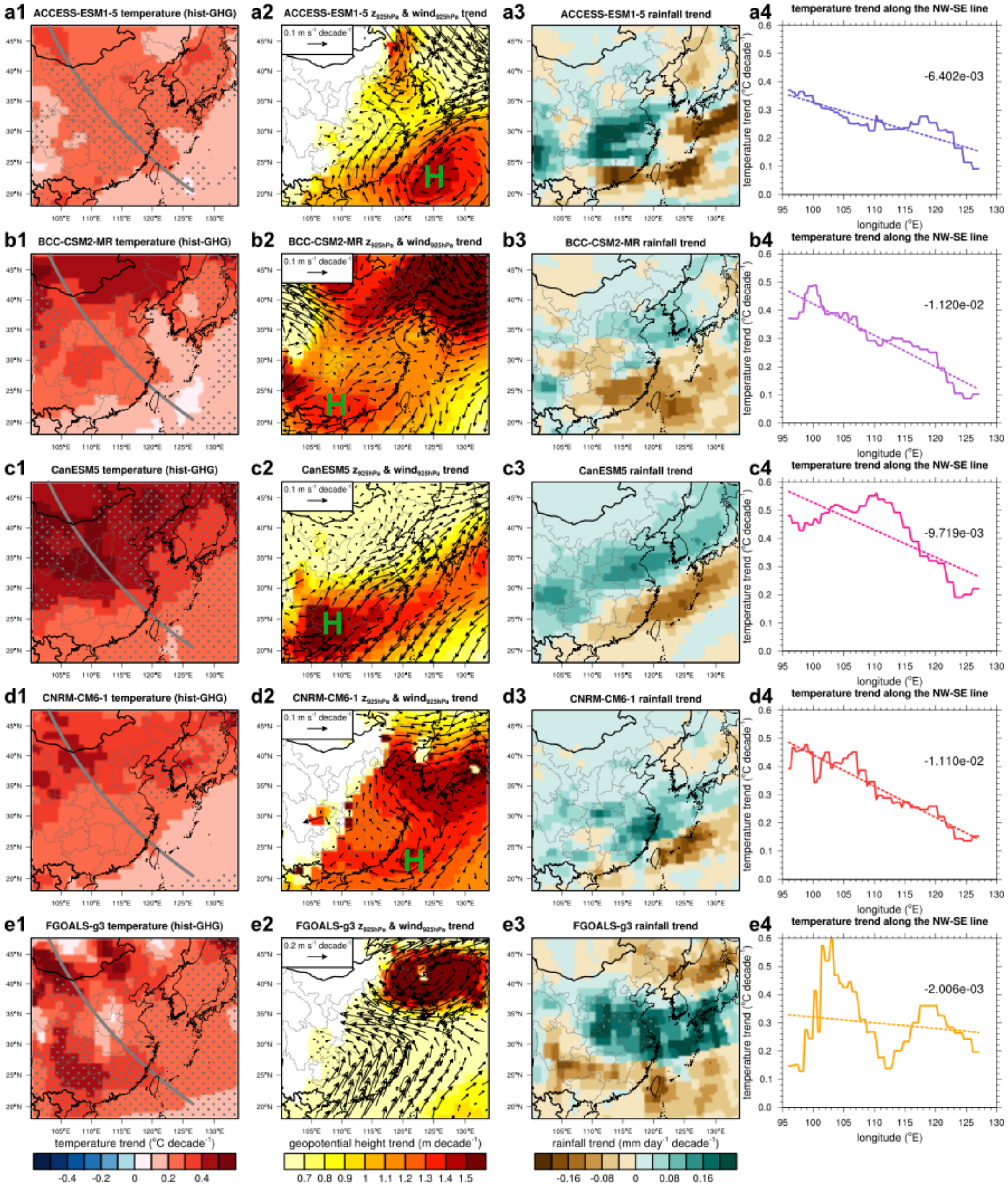

Supplementary Figure 6. Trends in temperature, geopotential height, wind, and rainfall for individual CMIP6 models (hist-GHG). (a1) Near-surface temperature trend during 1979–2020 for ACCESS-ESM1-5. (a2) Trends in geopotential height at 925 hPa and horizontal wind at 925 hPa. (a3) Rainfall trend. The stipples in (a1–a3) indicate that the trends are significant at the 90% confidence level (p-value < 0.1). (a4) Near-surface temperature trend along the gray

line marked in (a1). The dashed line is the linear regression line with respect to longitude, and the number indicates the slope of the regression line (unit is C° decade^{-1} degree^{-1}). Subfigures for (b) through (j) are the same as those for (a), but for a different model. The model name is indicated in the heading, and also can be found in Supplementary Table 1.

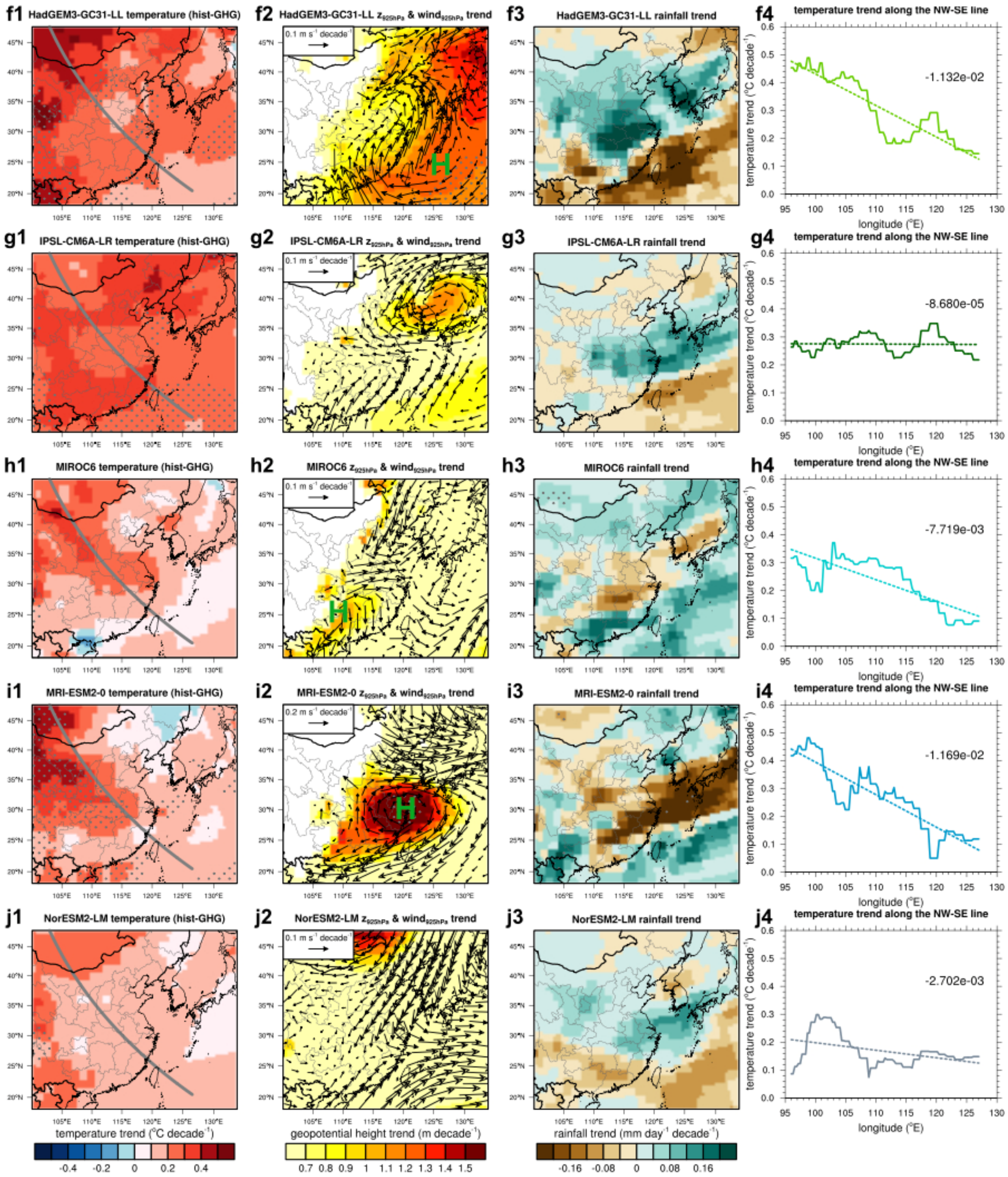

Supplementary Figure 6. (continue)

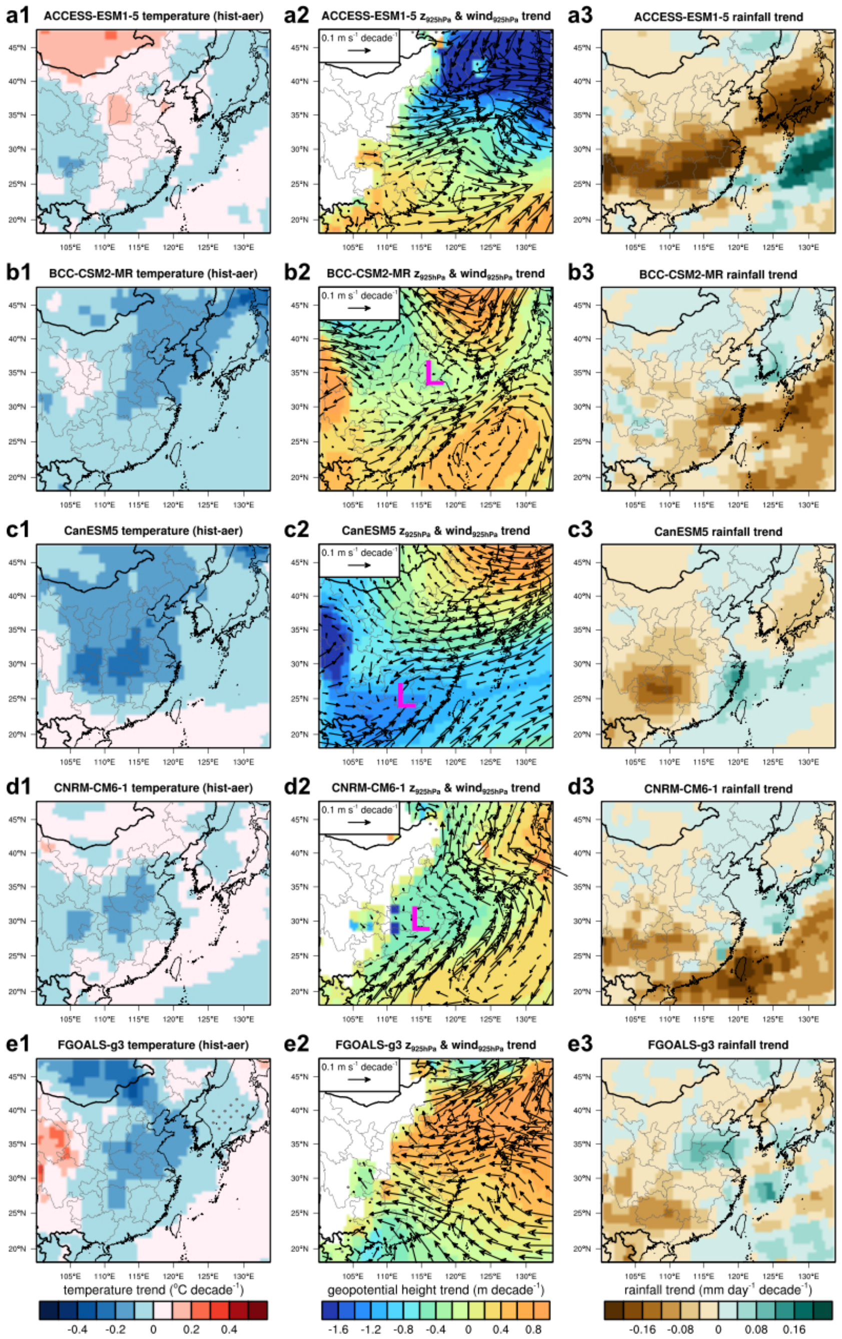

Supplementary Figure 7. Trends in temperature, geopotential height, wind, and rainfall for individual CMIP6 models (hist-aer). Same as the first through the third columns in Supplementary Fig. 6, but for CMIP6 hist-aer experiments.

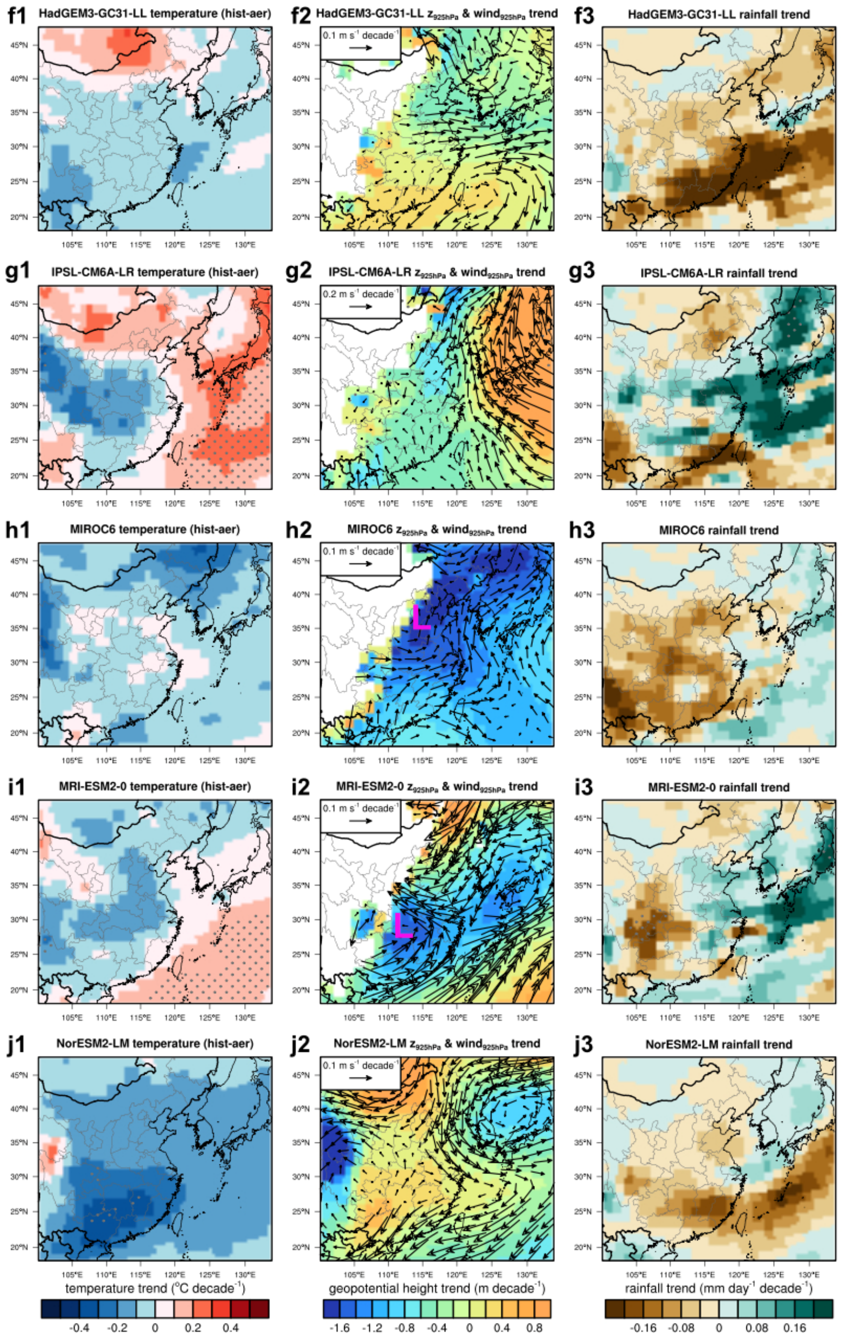

Supplementary Figure 7. (continue)

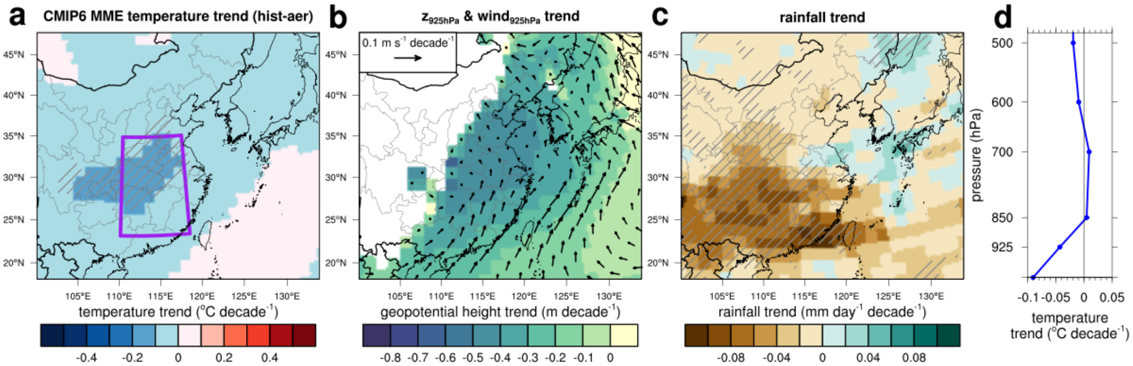

Supplementary Figure 8. Results for all 10 CMIP6 hist-aer experiments. Multi-model ensemble (MME) March-April mean (a) temperature trend, (b) geopotential height and winds at 925 hPa trends, (c) daily rainfall trend during 1979–2020, and (d) vertical profile of temperature trend averaged over central-east China (marked by the purple rectangle in a) in the CMIP6 hist-aer experiment. All of the 10 CMIP6 models are taken into account.

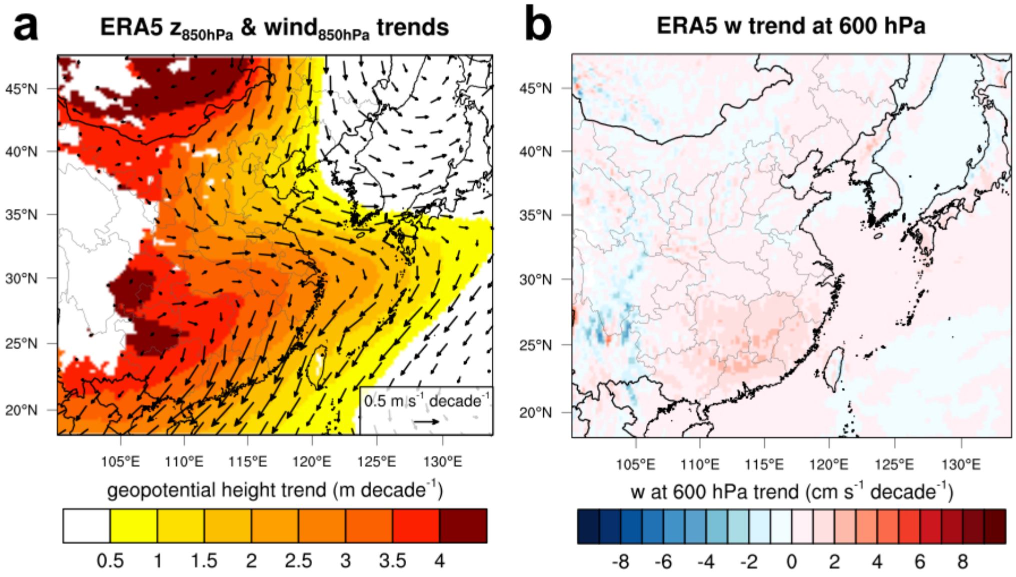

Supplementary Figure 9. Trends in 850-hPa geopotential height and wind and vertical velocity. ERA5 trends in March-April mean (a) geopotential height and horizontal winds at 850 hPa and (b) vertical velocity at 600 hPa during 1979–2020.

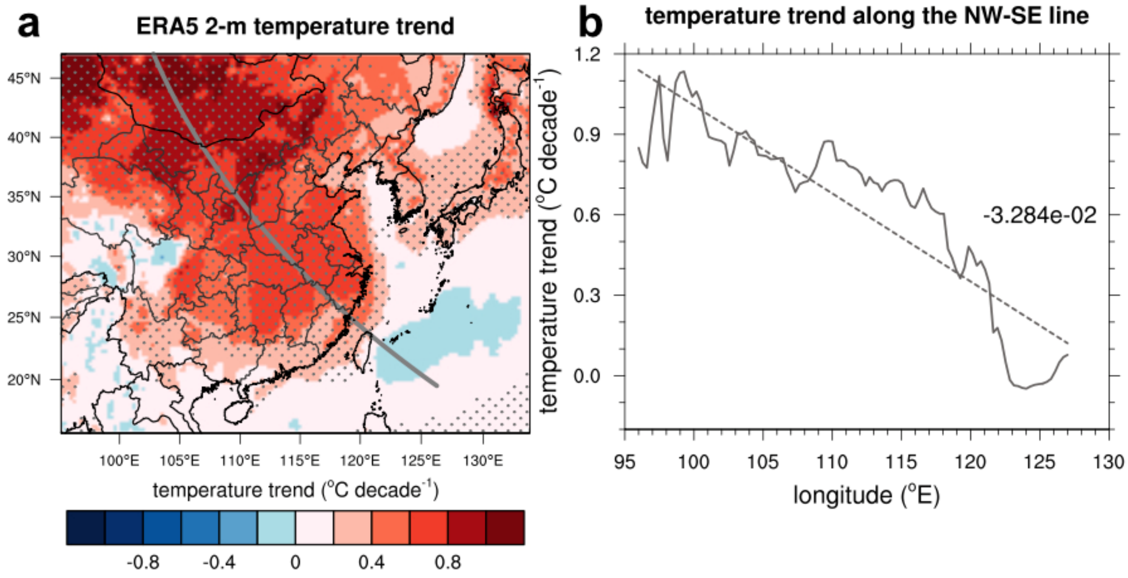

Supplementary Figure 10. Uneven temperature increasing trends. (a) ERA5 trends in March-April mean temperature at 2 m during 1979–2020. (b) 2-m temperature trend along the gray line in (a). The dashed line is the linear regression line with respect to longitude, and the number indicates the slope of the regression line (so the unit is C° decade^{-1} degree^{-1}).

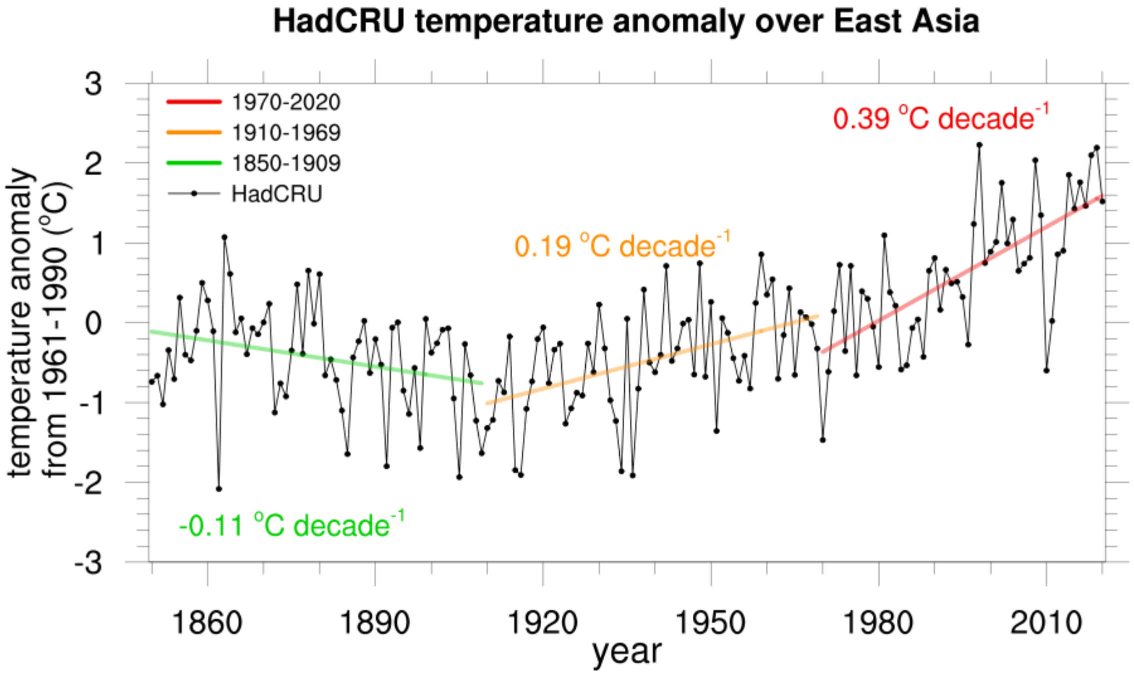

Supplementary Figure 11. Long-term temperature trends. Time series of HadCRUT5 temperature anomaly relative to 1961–1990 mean value averaged East Asia (18°–50°N, 105°–135°E). The March and April mean temperature for each year is shown here.

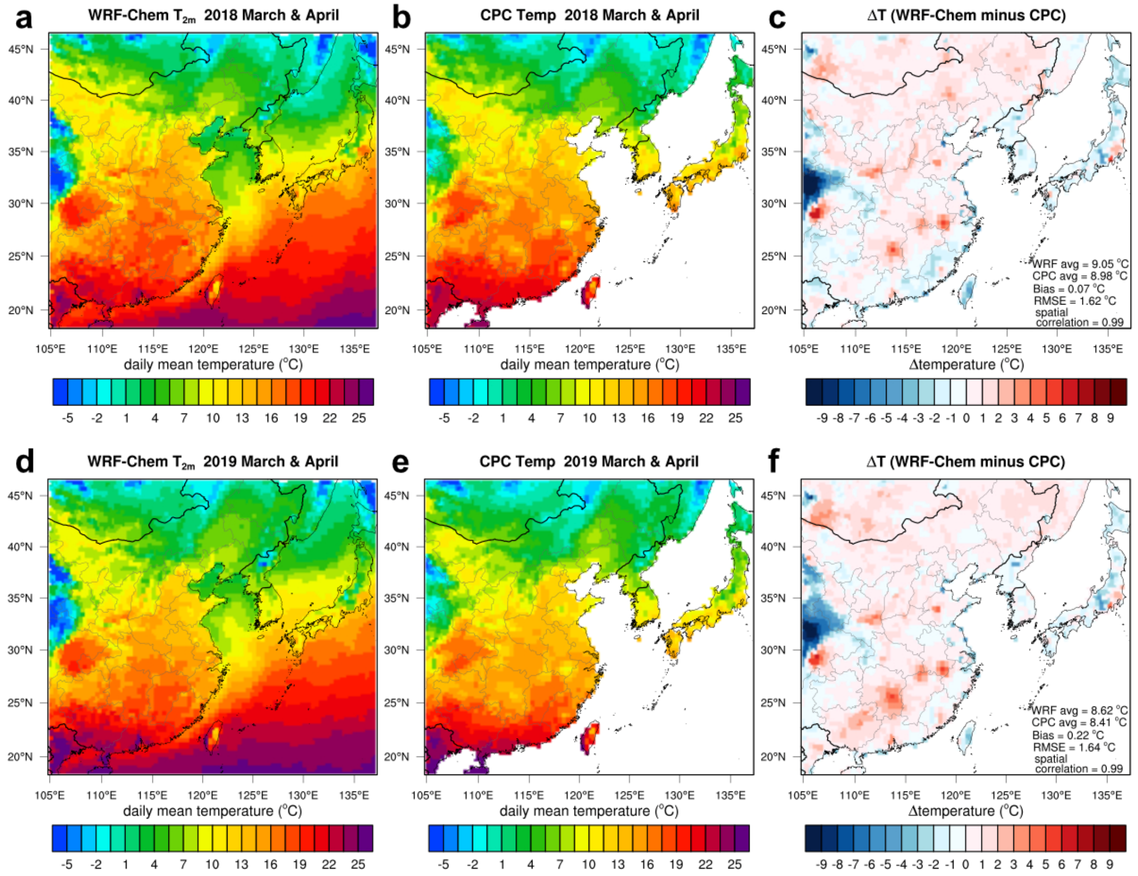

Supplementary Figure 12. Evaluation of WRF-Chem simulations for temperature. (a) March-April daily mean temperature at 2 m in the control WRF-Chem simulation (HIGH T_POLLUTED simulation) in 2018. (b) March-April 2018 daily mean temperature from the CPC gridded 2-m temperature data. (c) The difference in 2-m temperature between the simulated and observed data (simulated minus observed ones). The mean values from the WRF-Chem simulation and CPC data, mean bias error, root-mean-square-error, and spatial correlation averaged over the entire domain are shown in the right-bottom corner. (d–f) Same as in (a–c), respectively, but for March-April 2019.

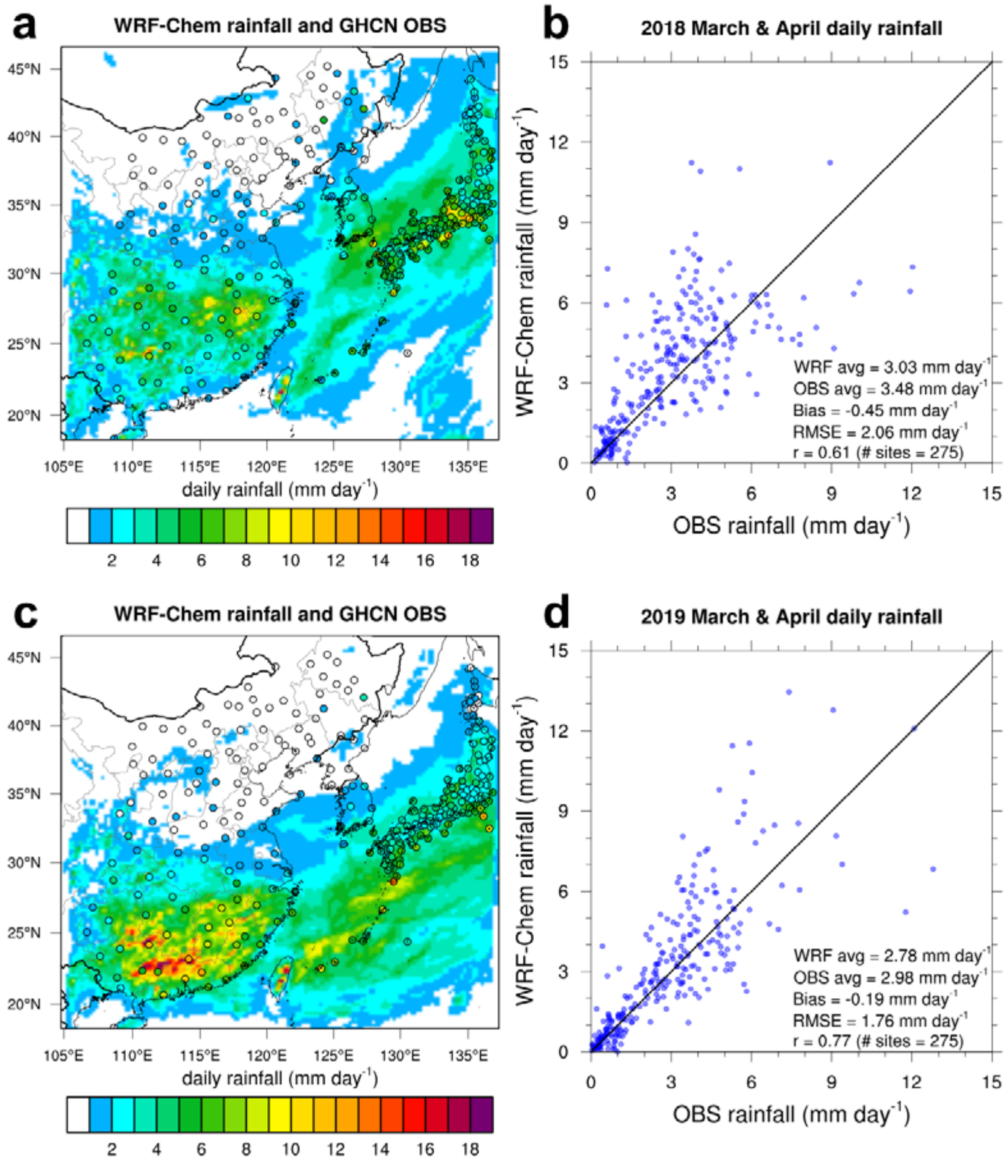

Supplementary Figure 13. Evaluation of WRF-Chem simulations for precipitation. (a) WRF-Chem simulated daily precipitation averaged during March-April 2018 (shaded) and GHCN station-observed daily precipitation (markers). (b) Scatter plot of observed and simulated precipitation. The mean values from the WRF-Chem simulation and GHCN data, mean bias error, root-mean-square-error, and linear correlation coefficients averaged over the stations are shown in the right-bottom corner. (c and d) Same as in (a and b), respectively, but for March-April 2019.

Supplementary References

1. Iacono, M. J. *et al.* Radiative forcing by long-lived greenhouse gases: Calculations with the AER radiative transfer models. *J. Geophys. Res.-Atmos.* **113**, D13103, (2008).
2. Nakanishi, M. & Niino, H. An Improved Mellor–Yamada Level-3 Model: Its Numerical Stability and Application to a Regional Prediction of Advection Fog. *Bound.-Layer Meteor.* **119**, 397–407 (2006).
3. Chen, F. & Dudhia, J. Coupling an Advanced Land Surface–Hydrology Model with the Penn State–NCAR MM5 Modeling System. Part I: Model Implementation and Sensitivity. *Mon. Wea. Rev.* **129**, 569–585 (2001).
4. Grell, G. A. & Freitas, S. R. A scale and aerosol aware stochastic convective parameterization for weather and air quality modeling. *Atmos. Chem. Phys.* **14**, 5233–5250 (2014).
5. Knote, C. *et al.* Simulation of semi-explicit mechanisms of SOA formation from glyoxal in aerosol in a 3-D model. *Atmos. Chem. Phys.* **14**, 6213–6239 (2014).
6. Knote, C., Hodzic, A. & Jimenez, J. L. The effect of dry and wet deposition of condensable vapors on secondary organic aerosols concentrations over the continental US. *Atmos. Chem. Phys.* **15**, 1–18 (2015).
7. Abdul-Razzak, H. & Ghan, S. J. A parameterization of aerosol activation 3. Sectional representation. *J. Geophys. Res.-Atmos.* ***107(D3)**, doi:10.1029/2001JD000483* (2002).
8. Fahey, K. M. & Pandis, S. N. Optimizing model performance: variable size resolution in cloud chemistry modeling. *Atmos. Environ.* **35**, 4471–4478 (2001).
9. Walcek, C. J. & Taylor, G. R. A Theoretical Method for Computing Vertical Distributions of Acidity and Sulfate Production within Cumulus Clouds. *J. Atmos. Sci.* **43**, 339–355 (1986).
10. Ryu, Y.-H., Hodzic, A., Barre, J., Descombes, G. & Minnis, P. Quantifying errors in surface

ozone predictions associated with clouds over the CONUS: a WRF-Chem modeling study using satellite cloud retrievals. *Atmos. Chem. Phys.* **18**, 7509–7525 (2018).

11. Wesely, M. L. Parameterization of surface resistances to gaseous dry deposition in regional-scale numerical models. *Atmos. Environ. (1967)* **23**, 1293–1304 (1989).
12. Binkowski, F. S. & Shankar, U. The Regional Particulate Matter Model: 1. Model description and preliminary results. *J. Geophys. Res.-Atmos.* **100**, 26191–26209 (1995).
13. Ryu, Y.-H. & Min, S.-K. Improving wet and dry deposition of aerosols in WRF-Chem: Updates to below-cloud scavenging and coarse-particle dry deposition. *J. Adv. Model. Earth Syst.* **14,** e2021MS002792 (2022).
14. Grell, G. A. & Devenyi, D. A generalized approach to parameterizing convection combining ensemble and data assimilation techniques. *Geophys. Res. Lett.* **29**, 38–1 (2002).
15. Inness, A. *et al.* The CAMS reanalysis of atmospheric composition. *Atmos. Chem. Phys.* **19**, 3515–3556 (2019).
16. Kurokawa, J. & Ohara, T. Long-term historical trends in air pollutant emissions in Asia: Regional Emission inventory in ASia (REAS) version 3. *Atmos. Chem. Phys.* **20**, 12761–12793 (2020).
17. Guenther, A. *et al.* Estimates of global terrestrial isoprene emissions using MEGAN (Model of Emissions of Gases and Aerosols from Nature). *Atmos. Chem. Phys.* **6**, 3181–3210 (2006).
18. Kaiser, J. W. *et al.* Biomass burning emissions estimated with a global fire assimilation system based on observed fire radiative power. *Biogeosciences* **9**, 527–554 (2012).
19. Rémy, S. *et al.* Two global data sets of daily fire emission injection heights since 2003. *Atmos. Chem. Phys.* **17**, 2921–2942 (2017).
20. Ginoux, P. *et al.* Sources and distributions of dust aerosols simulated with the GOCART model. *J. Geophys. Res.-Atmos.* **106**, 20255–20273 (2001).

21. Zhao, C. *et al.* The spatial distribution of mineral dust and its shortwave radiative forcing over North Africa: modeling sensitivities to dust emissions and aerosol size treatments. *Atmos. Chem. Phys.* **10**, 8821–8838 (2010).

22. Gong, S. L., Barrie, L. A. & Blanchet, J.-P. Modeling sea-salt aerosols in the atmosphere: 1. Model development. *J. Geophys. Res.-Atmos.* **102**, 3805–3818 (1997).
